# Relationship Between Receipt of 5,000 Ppm Sodium Fluoride Dentifrice Prescription and Time to First Dental Restoration among Older Adults: A Retrospective Cohort Study

**DOI:** 10.1111/scd.70151

**Published:** 2026-02-16

**Authors:** Rohit Nair, Oscar A. Rysavy, Leonardo Marchini, Daniel J. Caplan

**Affiliations:** ^1^ Department of Preventive and Community Dentistry The University of Iowa College of Dentistry and Dental Clinics Iowa City Iowa USA; ^2^ Division of Biostatistics and Computational Biology The University of Iowa College of Dentistry and Dental Clinics Iowa City Iowa USA

**Keywords:** dental caries, older adults, sodium fluoride

## Abstract

**Purpose:**

To evaluate the relationship between receipt of a 5,000 ppm sodium fluoride dentifrice prescription and time to first dental restoration in a population of older adults.

**Methods:**

Electronic dental records of patients aged ≥ 65 years who received a caries risk assessment at their initial comprehensive examination between 2009 and 2019 were analyzed to assess differences in the time to first restoration between patients who received a 5,000 ppm sodium fluoride dentifrice prescription and those who did not. Multivariable Cox regression was used to evaluate the time to first restoration after the caries risk assessment, controlling for covariates of interest.

**Results:**

The dataset included 3,741 participants, of whom 48% were women and 77% were self‐pay. In the final multivariable Cox regression model generated using the Bayesian Information Criterion for variable selection, having a 5,000 ppm sodium fluoride dentifrice prescription within 14 days after the caries risk assessment (HR 1.16, 95% CI 1.00–1.36), having 5 or more teeth with caries (HR 1.30, 95% CI 1.18–1.45), not having a dental home (HR 1.29, 95% CI 1.16–1.44), and eating > 3 snacks per day (HR 1.23, 95% CI 1.10–1.39), were the most important predictors of a faster time to first restoration.

**Conclusion:**

This study identified having a prescription for high‐fluoride dentifrice, number of teeth with caries, not having a dental home, and eating > 3 snacks per day as being associated with greater hazard of first restoration among older adult patients seeking comprehensive care in a dental school clinic.

## 1 Introduction

As the global population ages, an increasing number of older adults are retaining more of their natural teeth [1, 2] and experiencing greater gingival recession, leading to a heightened risk of both coronal and root caries [3, 4]. Recent data underscore the substantial burden of dental caries among older adults globally and in the United States. A 2025 systematic review and meta‐analysis of studies published between 1991 and 2024 estimated the global prevalence of dental caries among individuals aged 60 years and older to be 60.7% (95% CI: 54.6%–66.4%), with the highest prevalence observed in the Americas at 79.6% [5]. In the United States, the CDC's 2024 Oral Health Surveillance Report, based on NHANES data from 2017 to early 2020, found that nearly 96% of adults aged 65 years and older had experienced dental caries in their permanent teeth, and approximately 20% had untreated decay at the time of examination [6]. These findings highlight the persistent and widespread nature of dental caries in older populations and reinforce the need for targeted preventive strategies and improved access to dental care. Despite a decline in prevalence over recent decades in industrialized countries, the oral disease burden remains high within this demographic. [4, 7] This persistence is attributed to several prevalent risk factors, including polypharmacy (often resulting in xerostomia) [8], reduced dexterity [9], cognitive impairment [10], dependence on caregivers, [9] and financial constraints [11]. Additionally, the extended retention of natural teeth among older adults increases their susceptibility to dental caries [3, 12].

Beyond the oral implications, untreated caries and other oral diseases such as periodontal disease can have significant systemic consequences [13, 14]. Odontogenic infections from untreated caries can cause severe pain and potentially life‐threatening conditions [13]. Extensive caries and periodontal disease can also impair mastication, leading to malnutrition [15], and may exacerbate systemic conditions such as diabetes [16] and cardiovascular diseases [17]. This interconnectedness necessitates a comprehensive approach to healthcare that includes regular dental check‐ups and preventive measures adapted to the needs of older adults [18]. Therefore, it is key for oral healthcare providers to implement effective strategies to reduce the oral disease burden among older adults.

In this context, dental caries prevention remains a critical intervention for this population [19]. The prescription of 5,000 ppm sodium fluoride dentifrice has gained attention in the literature and is commonly recommended for high dental caries risk populations, such as older adults with complex health conditions. [19, 20] For that reason, evaluating the effectiveness of high‐fluoride dentifrices in various patient populations is important. Several published studies and systematic reviews have evaluated the effectiveness of 5000 ppm sodium fluoride (NaF) dentifrice in preventing and managing dental caries, particularly among high‐risk populations. A comprehensive systematic review on the anticariogenic efficacy of 5000 ppm fluoride dentifrice analyzed 14 studies and concluded that high‐fluoride dentifrice significantly reduces dental caries incidence, plaque accumulation, and bacterial counts (including mutans streptococci and lactobacilli) [21]. Additionally, it enhances remineralization and increases fluoride retention in both plaque and saliva.

Clinical trials have further supported these findings. For example, a randomized controlled trial by Ekstrand et al. involving elderly nursing home residents found that participants using 5000 ppm fluoride dentifrice (Duraphat) had three times more arrested root caries lesions after eight months compared to those using 1450 ppm fluoride dentifrice [22]. Similarly, Baysan et al. reported that 5000 ppm fluoride dentifrice resulted in 54.9% remineralization of root caries lesions, compared to only 16.2% in the 1100 ppm group [23]. Another study investigating the protective effects of high‐concentration fluoride solutions found that both 5000 ppm and 19,000 ppm NaF provided significantly better protection against enamel erosion and abrasion than standard 1450 ppm formulations [24]. The enhanced efficacy of high fluoride toothpaste is attributed to its ability to significantly elevate fluoride concentrations in saliva and dental plaque, which promotes remineralization and inhibits demineralization. This elevated fluoride environment also facilitates the formation of calcium fluoride reservoirs on tooth surfaces, serving as a sustained source of fluoride. Additionally, high fluoride levels modulate the oral microbiome by reducing cariogenic bacteria and plaque acidogenicity, thereby creating a less favorable environment for caries development.

These findings support the targeted use of high fluoride dentifrices as a preventive strategy for managing root caries in older adults and other high‐risk populations. While further research is needed to explore their effects on coronal caries in this demographic, current evidence strongly favors their use in clinical practice for root caries management. While some prospective studies have demonstrated its short‐term effectiveness [25, 26], this retrospective cohort study aimed to evaluate the relationship between receiving a prescription for a high‐fluoride dentifrice and time to first restoration in an older adult population.

## 2 Methods

In this retrospective study, electronic dental records were utilized to investigate time to first dental restoration among unique patients aged 65 or older who received a Caries Risk Assessment (CRA) at the University of Iowa College of Dentistry between 2009 and 2019. The Caries Risk Assessment used in this study was the standardized form developed by the American Dental Association (ADA) (see Supplemental Material). This form evaluates caries risk based on clinical conditions, environmental characteristics, and general health conditions, and was used for all patients included in the analysis. The inclusion criteria for this project were limited to charts of patients aged 65 years or older who had received a CRA during this period. This study was considered exempt from oversight by the University's institutional review board due to the de‐identified nature of the data (IRB ID# 202301151).

For all patients, baseline was the date three months after the CRA date in order to minimize the effect of restorations performed on teeth that already were carious at the time of the CRA. Follow‐up ended on the date of the first restoration after baseline (identified using the specific American Dental Association procedure codes shown in Table A in the Supplementary Material). If a patient did not have a restoration after baseline, follow‐up was censored on the date of the patient's last periodic recall examination on or before 12/31/22. Follow‐up time for each patient was calculated as the number of days from baseline until their restoration or censoring date. Given that the primary exposure variable was the prescription of high fluoride dentifrice, a 14‐day window following the CRA was established during which prescriptions had to be issued. This timeframe accounted for practical considerations such as delays due to faculty approval, missed prescriptions that required mailing, or other administrative factors that could prevent immediate issuance at the CRA appointment.

De‐identified patient‐level variables were collected at baseline. These included the patient's age, sex, insurance status, the responses to the CRA questions, self‐reported alcohol use, smoking, eating disorders, mental health issues, diabetes, number of medications, dental fear, dry mouth, bruxism, osteoarthritis, number of teeth, and number of teeth with at least one carious lesion. A complete list of variables is shown in Tables 1a–1c.

**TABLE 1a:** Baseline characteristics of the study cohort (*n* = 3,741):From electronic dental record (7 variables).

	Dentifrice prescription	
Variable	No, *N* = 3414	Yes, *N* = 327
Age (Years)		
65–69	1,069 (31%)	94 (29%)
70–79	1,653 (48%)	166 (51%)
80 +	692 (20%)	67 (20%)
GENDER		
Female	1,645 (48%)	159 (49%)
Male	1,769 (52%)	168 (51%)
INSURANCE		
Private	730 (21%)	81 (25%)
Self Pay	2,639 (77%)	242 (74%)
XIX/DWP (Gov't Funding)	45 (1.3%)	4 (1.2%)
Medications		
0–2	711 (21%)	38 (12%)
3–5	1,030 (30%)	77 (24%)
6–10	1,084 (32%)	126 (39%)
11 +	589 (17%)	86 (26%)
Number of Teeth		
2–16	508 (15%)	54 (17%)
17–24	1,190 (35%)	146 (45%)
25 +	1,716 (50%)	127 (39%)
Number of Teeth with Caries		
0	1,375 (40%)	106 (32%)
1–4	1,215 (36%)	123 (38%)
5 +	824 (24%)	98 (30%)

**TABLE 1b:** Baseline characteristics of the study cohort (*n* = 3,741):From health history (9 variables).

Variable	Yes	No	Missing
Do you drink alcoholic beverages?	539 (14%)	699 (19%)	2,503 (67%)
Do you or have you used tobacco?	752 (20%)	1,049 (28%)	1,940 (52%)
Eating disorder (Bulimia, Anorexia, other)?	6 (0.2%)	1,167 (31%)	2,568 (69%)
Mental health issues (Bipolar disorder, Depression, Schizophrenia, PTSD, ADD/ADHD, Generalized anxiety disorder, Panic attacks, other)?	379 (10%)	3,337 (89%)	25 (0.7%)
Diabetes (Type I, II, other)?	787 (21%)	2,933 (78%)	21 (0.6%)
Osteoarthritis?	1,115 (30%)	2,232 (60%)	394 (10%)
Has fear ever prevented you from seeking dental care?	392 (10%)	3,185 (85%)	162 (4.4%)
Do you experience dry mouth?	576 (15%)	2,100 (56%)	1,065 (28%)
Do you clench, brux, or grind your teeth?	179 (4.8%)	625 (17%)	2,937 (78%)

**TABLE 1c:** Baseline characteristics of the study cohort (*n* = 3,741): From Caries Risk Assessment (15 variables).

Variable	Yes	No	Missing
Fluoride Exposure (through drinking water, supplements, professional applications, toothpaste)	2,631 (70%)	358 (9.6%)	752 (20%)
Dental Home: Established patient of record, receiving regular dental care in a dental office	2,183 (58%)	770 (21%)	788 (21%)
Eats more than 3 meals per day	638 (17%)	2,379 (64%)	724 (19%)
Eats more than 3 snacks per day	541 (14%)	2,361 (63%)	839 (22%)
Meals/snacks are not structured (on and off—grazing)	830 (22%)	2,163 (58%)	748 (20%)
Drinks sugared beverages (juice, soft drinks, energy drinks) daily	654 (17%)	2,284 (61%)	803 (21%)
Drinks more than 20oz of sugared beverages daily	244 (7%)	2,622 (70%)	875 (23%)
Drinks beverages for more than 30 min per day	351 (9%)	2,527 (68%)	863 (23%)
Eats sugared candy or medicated lozenges daily	517 (14%)	2,391 (64%)	833 (22%)
Special Health Care Needs (developmental, physical, medical or mental disabilities that prevent or limit performance of adequate oral health care by themselves or caregivers) Low—No / Moderate /High‐ Yes	183 (5%)	2,665 (71%)	893 (24%)
Chemo/Radiation therapy (High—Yes / Low—No)	201 (5%)	2,660 (71%)	880 (24%)
Eating disorders (Low—No / Moderate—Yes)	14 (1%)	2,822 (75%)	905 (24%)
Smokeless tobacco use (Low—No / Moderate—Yes)	47 (1%)	2,794 (75%)	900 (24%)
Medications that reduce salivary flow (Low—No / Moderate—Yes)	1,271 (34%)	1,678 (45%)	792 (21%)
Drug/alcohol abuse (Low—No / Moderate—Yes)	54 (1%)	2,769 (74%)	918 (25%)

Descriptive statistics were used to characterize the cohort, with numeric variables binned into categories. Multivariable Cox proportional hazards models then were generated to analyze the effect of covariates on time to the first restorative intervention [27]. First, a full model was fitted that contained all the variables shown in Tables 1a–1c. Next, backward selection using the Bayesian Information Criterion (BIC) [28] was employed on the complete cases to select a final model, with the variable “prescription for high‐fluoride dentifrice” forced into the model. This model was subsequently fitted on all available data for the selected variables. In both models, scaled Schoenfeld residuals were used to evaluate adherence to the proportional hazards assumption. Analysis was performed in R version 4.4.1 using the package survival version 3.6–4. [29, 30, 31]

## 3 Results

The dataset included 3,741 participants, and the univariate distributions for the 31 variables analyzed are presented in Tables 1a–1c. Females comprised 48% of the sample, and the majority was self‐pay (77%), with 22% having private insurance and 1.3% having government funding. About a third were age 65–69, about half were age 70–79, and the remainder were age 80 +. The majority (62%) were taking between 3–10 medications. Almost 9% of the participants were prescribed a 5,000 ppm sodium fluoride dentifrice upon or within 14 days of receiving the CRA (Table 1b).

Regarding dental variables at baseline: Just under half of the participants had at least 25 remaining teeth, and about 40% had no teeth with caries. The majority of participants (70%) said they were exposed to low fluoride, and 58% reported having a dental home. Other notable health behaviors included 17% eating > 3 meals per day, 14% eating > 3 snacks per day, and 17% consuming sugary drinks daily.

Due to missing data in some of the predictor variables, the full multivariable Cox regression model contained *N* = 1,493 participants (model not shown). The final model contained four variables and used data from 2,736 (73%) of the participants. Table 2 shows the hazard ratios (HR), 95% confidence intervals, and *p*‐values for the final model. Having a 5,000 ppm sodium fluoride dentifrice prescription within 14 days after the CRA (HR 1.16, 95% CI 1.00–1.36) indicates an estimated 16% greater hazard of first restoration at any point in the study compared to those without such a prescription. Other variables in the model were having 5 or more teeth with caries (HR 1.30, 95% CI 1.18–1.45), not having a dental home (HR 1.29, 95% CI 1.16–1.44), and eating > 3 snacks per day (HR 1.23, 95% CI 1.10–1.39).

**TABLE 2:** Final Cox Regression Model (*N* = 2,736).

Variable	HR	95% CI	*p*‐value
Prescription for high‐fluoride dentifrice			
No	—	—	
Yes	1.16	1.00, 1.36	0.051
Number of teeth with caries			
0–4	—	—	
5 +	1.30	1.18, 1.45	< 0.001
Dental Home			
Yes	—	—	
No	1.29	1.16, 1.44	< 0.001
Eats > 3 snacks per day			
No	—	—	
Yes	1.23	1.10, 1.39	< 0.001

## 4 Discussion

The findings of this retrospective cohort study provide some perspective into the relationship between 5,000 ppm sodium fluoride dentifrice prescription and time to first dental restoration among older adults. The final Cox regression model identified three additional factors for a faster time to first dental restoration: baseline number of teeth with caries, not having a dental home, and eating > 3 snacks per day.

A previous study found that while over two‐thirds of dentists administered caries risk assessments (CRAs) in some form, less than 20% used a formal written questionnaire. Recent graduates, dentists who preferred non‐surgical treatment for early lesions, and those in large group practices were more likely to use CRAs [32]. Training students in CRA tools was linked to the successful adoption of risk‐based, non‐operative dental caries management programs [33].

The American Dental Association Caries Risk Assessment Form collects information on various factors to assess dental caries risk as “low,” “moderate,” or “high.” However, it is not prescriptive, and dentists should use clinical judgment to determine caries risk based on the patient's overall health and social situation. Consequently, the overall caries risk classification was not captured for this analysis, as it could vary based on individual providers’ interpretations of additional factors. Including this variable could have introduced a misclassification bias in the results. In clinical practice, an assessment of “moderate” or “high” dental caries risk is likely to prompt providers to prescribe as prescription‐strength fluoride dentifrice.

Older adults are widely recognized as a high‐risk group for the development of dental caries due to a combination of age‐related physiological changes and lifestyle factors. A recent National Health and Nutrition Examination Survey reported that 20.2% of U.S. adults aged 65 and older had some degree of untreated dental caries [34]. Globally, oral health conditions such as edentulism, partial edentulism, and untreated dental caries remain highly prevalent in this age group. Untreated dental caries in permanent teeth remains the most prevalent oral condition worldwide, affecting 27.5% of the global population [35], with estimates for older adults being as high as 60.7% [5].

Local factors that lead to enhanced risk include age or polypharmacy related reduction in salivary flow, diminished salivary buffering capacity, gingival recession that exposes root surfaces, etc. Additionally, physical or cognitive impairments may limit the ability to maintain effective oral hygiene. To compensate for masticatory deficiencies, dietary patterns in older adults may shift toward softer, carbohydrate‐rich foods, further increasing caries risk. Many individuals in this age group also have existing dental restorations or wear removable prostheses, which without adequate hygiene can harbor cariogenic bacteria and contribute to recurrent dental caries. Collectively, these factors contribute to the elevated burden of dental caries observed in older adult populations [36].

Untreated dental caries is a major contributor to tooth loss and, in severe cases, can lead to complete edentulism. Reports suggest a global age‐standardized prevalence of edentulism at 4.11%, with the highest burden in those aged 70+ [37]. Partial edentulism is more common, often resulting in fewer than 20 teeth, and retention rates vary widely by region and socioeconomic status. In the US, adults aged 65 years and over retained 20.7 teeth on average with 13.8% of this population experiencing complete tooth loss.

Although our study indicated that prescribing high‐fluoride dentifrice to a high‐risk group was associated with a higher hazard of restoration placement, it is important to consider that high‐fluoride dentifrice was likely prescribed to patients deemed to have the highest risk of developing dental caries in the first place, potentially introducing significant bias. This association likely reflects confounding by indication wherein providers may have selectively prescribed high‐fluoride dentifrice to patients they perceived as being at elevated risk for caries and consequently more likely to need restorations. Importantly, in this study, the Caries Risk Assessment (CRA) score or risk category was not directly linked to the prescription event. The CRA was used solely as a criterion to identify a cohort of older adults (aged 65 and above) for inclusion in the study. Approximately 9% of patients who received a CRA were prescribed high‐fluoride dentifrice, but we cannot determine whether these individuals were formally categorized as “high risk” because that variable was not captured in the dataset. This limits our ability to assess whether preventive care was equitably distributed among those who may have benefited most. Our findings underscore the need for more consistent documentation of caries risk and clearer protocols for preventive fluoride dentifrice prescriptions in older adult populations. Additionally, we were unable to track patient adherence, so it is possible that some patients may not have filled their prescriptions, and even if they did, they might not have used the dentifrice as prescribed. It is also possible that patients who were given a prescription fluoride dentifrice might be more receptive to getting restorations because they might be at higher risk for dental caries, especially around existing restorations.

People with lower dental caries risk might not feel the need to rush restorative care especially if it is determined that they will be able to adhere to an individualized plan for early dental caries lesion management. From a logistical perspective, it is also possible that those who received a prescription were also the ones who had more time available to come to appointments at the times offered by the dental school.

In this study, patients with more than five carious lesions at when the CRA was performed had an estimated 30% greater hazard of restoration than those with fewer lesions. This is not surprising, as previous dental caries experience is a known risk factor for new dental caries lesions in older adults, particularly on root surfaces [12, 38]. Another study found that higher adherence to healthy dietary patterns was associated with greater odds of healthy aging, including cognitive, physical, and mental health, and living to 70 years of age free of chronic diseases [39]. While the study did not address meal frequency, higher intakes of fruits, vegetables, whole grains, unsaturated fats, nuts, legumes, and low‐fat dairy were linked to better aging outcomes. Conversely, higher intakes of trans fats, sodium, sugary beverages, and other processed foods were negatively associated with healthy aging. Without distinguishing between cariogenic and non‐cariogenic foods, our results indicated that the frequency of snacks consumed each day contributed to the likelihood of needing restorative dental work. Consuming more than three cariogenic snacks per day would likely be more detrimental to oral health than consuming healthy snacks at the same rate [40]. On the other hand, healthy snack options may be more fibrous and tougher to chew compared to the softer consistency of foods that are high in refined carbohydrates. Consequently, increased frequency of snacking among older adults and the resulting elevated cyclic masticatory loads, could lead to sudden mechanical failures thereby creating new restorative needs.

Nevertheless, our study represents a first step in elucidating the effect of high‐fluoride dentifrice on the time‐to‐first dental restoration in older adult populations. The continued use of 5,000 ppm sodium fluoride dentifrice is a relatively inexpensive preventive measure that has been widely recommended to high dental caries risk groups, particularly older adults, due to factors such as polypharmacy, reduced dexterity, and cognitive impairment [12, 20, 25, 26]. Our results suggest that prescribing a high‐fluoride dentifrice may reflect provider recognition of elevated risk, but additional preventive measures may be necessary to effectively reduce restorative burden in this group. For older adults with these characteristics and a heavily restored dentition, there is a need for customized treatment strategies, which might include an even higher exposure to topical fluoride, for example, through professional application of silver diamine fluoride and fluoride varnish [19]. Reducing exposure to cariogenic foods in the diet, regular dental check‐ups and maintaining a dental home might be some other strategies to improve tooth survival, especially for older adults with an extensively restored dentition [18, 19].

Our results also highlight the effect that a stable dental home has on older adult patients’ restorative needs. Low utilization of preventive services increases the risk of dental caries, periodontal disease and tooth loss in this patient group 41]. Older adults can benefit from having a consistent dental care provider, as a customized dental caries management plan can be better implemented by ensuring continuity of care [19]. Although using prescription fluoride dentifrice is a key preventive measure, it is equally important to ensure that brushing is performed in a timely manner. Some studies have shown that the benefit of twice daily brushing with prescription strength fluoride dentifrices diminishes with advancing age, as the presence of visible, heavy plaque deposits contributes to higher dental caries risk for older adults [12, 42]. Any protection that such dentifrices offer this cohort could be augmented by educating patients on effective ways for mechanical plaque removal including the use of electric toothbrushes with ergonomic handle designs that allow users to overcome difficulties with brushing resulting from poor manual dexterity [19]. Training nursing aides at long term care facilities to properly brush dependent residents’ teeth has been documented to reduce plaque scores 43]. Older adults are also at risk for dry mouth resulting from varying etiologies, most commonly polypharmacy and dehydration [8]. Reducing dental caries activity for this group would also entail measures to promote oral hydration and the use of saliva substitutes. [18, 19] Our findings reinforce the importance of improving access to regular dental care for older adults, particularly those with limited mobility or financial constraints 44]. Although the majority of participants in our study were relatively healthy and functionally independent—with only a small proportion reporting special care needs—the broader implications of our findings may still inform preventive strategies for more care‐dependent older adults, who often face greater challenges in maintaining oral health due to physical, cognitive, or socioeconomic limitations.

This study has several limitations. The retrospective design may introduce selection bias, as the sample was limited to patients who sought care at a specific dental clinic. Additionally, reliance on electronic dental records may result in incomplete data, particularly for self‐reported variables such as dietary habits and medical conditions. Another consequence of incomplete data is the inability to identify whether new restorations were due to new carious lesions (i.e., observed after the CRA), meaning some restorations might not have been preventable with high fluoride dentifrice. The three‐month cutoff for defining baseline was based on institutional norms around treatment timelines, including clinical rotation schedules and typical care delivery pace. While practical, this timeframe does not guarantee that all planned treatment was completed by baseline, which is a limitation. We were also unable to determine the specific clinical reason for the first restoration placed after baseline—whether due to primary caries, recurrent caries, tooth fracture, or restoration failure. Although diagnostic codes and chart entries potentially could have provided this detail, they were not included in the data extraction, and inconsistent charting practices further limited reliable classification. The generalizability of these findings is also limited to similar populations and settings, necessitating further research to confirm these results in diverse cohorts.

Future research should focus on prospective studies with larger and more diverse older adult populations to validate these findings. Additionally, exploring the long‐term effects of high‐fluoride dentifrice in conjunction with other preventive measures, such as dietary counseling and regular dental check‐ups, could provide a more comprehensive understanding of dental caries prevention in older adults. Investigating the cost‐effectiveness of these interventions would also be valuable for informing healthcare policy and ensuring that effective preventive measures are accessible to all older adults. These findings could inform clinical guidelines and enhance the quality of care provided to this vulnerable population, ultimately improving their oral and overall health outcomes.

## 5 Conclusion

The results of this retrospective cohort study identified prescription of 5,000 ppm sodium fluoride dentifrice, baseline number of teeth with caries, not having a dental home, and eating > 3 snacks per day as significant factors associated with a greater hazard of restoration events after the provision of a formal caries risk assessment.

## Authors Contribution

RN, LM, and DJC contributed to the conception and design of the study. RN, OAR and DJC contributed to acquisition, analysis, and interpretation of data. All authors approved this submitted version of the manuscript.

## Declaration of Generative AI and AI‐assisted Technologies in the Writing Process

In preparing this work, the authors employed Microsoft's AI language model, Co‐Pilot, to enhance the manuscript's clarity and language. Following its use, the authors carefully reviewed and revised the content as necessary, assuming full responsibility for the final published article.

## Supporting information



Supplementary Material
