# Supplementary material for: Relationship Between Receipt of 5,000 Ppm Sodium Fluoride Dentifrice Prescription and Time to First Dental Restoration among Older Adults: A Retrospective Cohort Study
Source: Spec Care Dentist. 2026 Feb 16;46(1):e70151. doi: 10.1111/scd.70151 (PMC12908106; doi:10.1111/scd.70151)
Supplement: Supplementary file 1 — Supplementary Material [file SCD-46-0-s001.docx]

**Supplementary Material**

**Table A.** List of exam and restorative treatment procedural codes retrieved from subsequent appointments after prescription.

| **Preventive Codes** |
| --- |
| Examination: D0120, D0120.6, D0120.7, D0150, D0150.2 |
| **Non-Preventive Codes** |
| Glass Ionomer type restorations: D2330.1, D2331.1, D2332.1, D2335.1, D2391.1, D2392.1, D2393.1, D2394.1, D2330.1, D2331.1, D2332.1, D2335.1, D2391.1, D2392.1, D2393.1, D2394.1, D2330.2, D2331.2, D2391.2, D2392.2  Resin composite type restorations: D2330, D2331, D2332, D2332.1, D2335, D2390, D2391, D2392, D2393, D2394, D2394.2, D2330.2, D2331, D2391, D2392, D2990  Sedative type restorations: D2940, D2940.1, D2940.2, D2940.3  Amalgam type restorations: D2140, D2150, D2160, D2161  Inlay/Onlay type restorations: D2510, D2520, D2530, D2543, D2544, D2610, D2620, D2630, D2642, D2642.5, D2643, D2643.5, D2644, D2644.5, D2650, D2651, D2652, D2662, D2662.5, D2663, D2663.5, D2664, D2664.5  Crowns: D2337, D2710, D2720, D2721, D2722, D2740, D2740.5, D2750, D2750.1, D2751, D2751.1, D2752, D2752.1, D2753, D2780, D2781, D2782, D2790, D2790.1, D2790.8, D2791, D2791.1, D2792, D2792.1  Veneers: D2960, D2961, D2962  Prefabricated crown: D2928, D2931, D2932, D2933, D2954 |

**Item 2:** ADA Caries Risk Assessment Form (Age >6 years). Source: American Dental Association. (2023, July 18). Caries risk assessment and management. ADA Library & Archives. Retrieved April 28, 2025, from https://www.ada.org/resources/ada-library/oral-health-topics/caries-risk-assessment-and-management


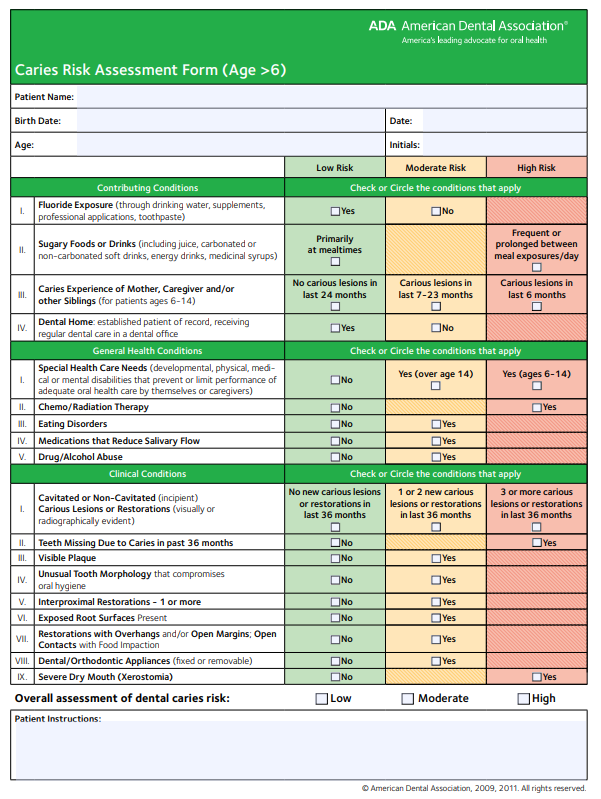


**Item 3:** Full Cox Regression Model

| **Characteristic** | **HR** | **95% CI** |
| --- | --- | --- |
| Prescription for high-fluoride  dentifrice |  |  |
| No | — | — |
| Yes | 1.26 | 1.01, 1.58 |
| Age (Years) |  |  |
| 65-69 | — | — |
| 70-79 | 0.94 | 0.82, 1.09 |
| 80+ | 1.05 | 0.87, 1.27 |
| Gender |  |  |
| Female | — | — |
| Male | 0.99 | 0.87, 1.13 |
| Insurance |  |  |
| INS | — | — |
| Self Pay | 0.94 | 0.80, 1.10 |
| XIX/DWP (Gov’t Funding) | 1.43 | 0.87, 2.37 |
| Medications |  |  |
| 0-2 | — | — |
| 3-5 | 1.06 | 0.88, 1.28 |
| 6-10 | 0.96 | 0.79, 1.18 |
| 11+ | 1.02 | 0.81, 1.30 |
| Number of teeth |  |  |
| 25+ | — | — |
| 2-16 | 0.85 | 0.70, 1.03 |
| 17-24 | 1.13 | 0.98, 1.30 |
| Number of teeth with caries |  |  |
| 0-4 | — | — |
| 5+ | 1.39 | 1.21, 1.60 |
| Fluoride exposure |  |  |
| Low - Yes | — | — |
| Moderate - No | 1.10 | 0.89, 1.36 |
| Dental home |  |  |
| Low - Yes | — | — |
| Moderate - No | 1.22 | 1.05, 1.41 |
| Eats more than 3 meals per day |  |  |
| No | — | — |
| Yes | 1.18 | 0.99, 1.40 |
| Eats more than 3 snacks per day |  |  |
| No | — | — |
| Yes | 1.31 | 1.09, 1.57 |
| Meals/snacks are not structured |  |  |
| No | — | — |
| Yes | 1.01 | 0.87, 1.19 |
| Drinks sugared beverages daily |  |  |
| No | — | — |
| Yes | 1.04 | 0.87, 1.24 |
| Drinks more than 20 oz of sugared  beverages daily |  |  |
| No | — | — |
| Yes | 1.11 | 0.84, 1.46 |
| Drinks beverages for more than 30  minutes per day |  |  |
| No | — | — |
| Yes | 1.13 | 0.90, 1.42 |
| Eats sugared candy or medicated  lozenges daily |  |  |
| No | — | — |
| Yes | 1.02 | 0.85, 1.22 |
| Special health care needs |  |  |
| Low | — | — |
| Mod/High | 1.12 | 0.85, 1.47 |
| Chemo / radiation therapy |  |  |
| Low - No | — | — |
| High - Yes | 1.09 | 0.85, 1.41 |
| Smokeless tobacco use |  |  |
| Low - No | — | — |
| Moderate - Yes | 0.88 | 0.54, 1.44 |
| Medications that reduce  salivary flow |  |  |
| Low - No | — | — |
| Moderate - Yes | 1.02 | 0.88, 1.19 |
| Drug / Alcohol use |  |  |
| Low - No | — | — |
| Moderate - Yes | 1.38 | 0.86, 2.19 |
| Mental health issues |  |  |
| No | — | — |
| Yes | 0.93 | 0.74, 1.16 |
| Diabetes |  |  |
| No | — | — |
| Yes | 1.04 | 0.87, 1.23 |
| Has fear ever prevented you from  seeking dental care? |  |  |
| No | — | — |
| Yes | 1.00 | 0.82, 1.22 |
| Do you experience dry mouth? |  |  |
| No | — | — |
| Yes | 1.05 | 0.89, 1.23 |
| Osteoarthritis |  |  |
| No | — | — |
| Yes | 1.06 | 0.92, 1.22 |
| Abbreviations: CI = Confidence Interval, HR = Hazard Ratio | | |
